# In-Season Yield Prediction of Cabbage with a Hand-Held Active Canopy Sensor

**DOI:** 10.3390/s17102287

**Published:** 2017-10-08

**Authors:** Rongting Ji, Ju Min, Yuan Wang, Hu Cheng, Hailin Zhang, Weiming Shi

**Affiliations:** 1 State Key Laboratory of Soil and Sustainable Agriculture, Institute of Soil Science, Chinese Academy of Sciences, Nanjing 210008, China; rtji@issas.ac.cn (R.J.); jmin@issas.ac.cn (J.M.); wangyuan@issas.ac.cn (Y.W.); hucheng@issas.ac.cn (H.C.); 2 University of Chinese Academy of Sciences, Beijing 100049, China; 3 Department of Plant and Soil Sciences, Oklahoma State University, Stillwater, OK 74078-6028, USA; hailin.zhang@okstate.edu

**Keywords:** Greenseeker, cabbage, NDVI, yield prediction, CGDD

## Abstract

Efficient and precise yield prediction is critical to optimize cabbage yields and guide fertilizer application. A two-year field experiment was conducted to establish a yield prediction model for cabbage by using the Greenseeker hand-held optical sensor. Two cabbage cultivars (Jianbao and Pingbao) were used and Jianbao cultivar was grown for 2 consecutive seasons but Pingbao was only grown in the second season. Four chemical nitrogen application rates were implemented: 0, 80, 140, and 200 kg·N·ha^−1^. Normalized difference vegetation index (NDVI) was collected 20, 50, 70, 80, 90, 100, 110, 120, 130, and 140 days after transplanting (DAT). Pearson correlation analysis and regression analysis were performed to identify the relationship between the NDVI measurements and harvested yields of cabbage. NDVI measurements obtained at 110 DAT were significantly correlated to yield and explained 87–89% and 75–82% of the cabbage yield variation of Jianbao cultivar over the two-year experiment and 77–81% of the yield variability of Pingbao cultivar. Adjusting the yield prediction models with CGDD (cumulative growing degree days) could make remarkable improvement to the accuracy of the prediction model and increase the determination coefficient to 0.82, while the modification with DFP (days from transplanting when GDD > 0) values did not. The integrated exponential yield prediction equation was better than linear or quadratic functions and could accurately make in-season estimation of cabbage yields with different cultivars between years.

## 1. Introduction

Cabbage (*Brassica oleracea* var. capitata) is recognized as a prominent source of mineral nutrition, sugar and vitamin C, and has been widely planted in China, Japan, USA, Korea, India and other countries [1,2]. The FAO reported that the world production of cabbage and other brassicas reached 70 million metric tons in 2012, with half of these crops grown in China for an area of 980,000 ha [3]. Southeastern China is one of the most intensive agricultural regions and the main producing areas of vegetable, with cabbage as one of the most popular and representative open-field vegetables over the winter. In fact, as a commercial cash crop, the yield of cabbage is the most common indicator and closely related to vegetable farmers’ profit [4]. In addition, cabbage yield is determined by many factors, e.g., fertilizer application rates, irrigation regimes, and environmental conditions, etc. [5,6,7]. As a common practice, ample nitrogen (N) fertilizer input is the predominant way to guarantee crop yields and maintain soil fertility [8]. However, the overuse or misuse of N fertilizer has influenced the soil quality and resulted in significant yield decrease of crops in many regions [9,10,11]. In this sense, yield monitoring has become an important aspect of N management scheme for cabbage production. In the past, researchers estimated cabbage yield by counting the head number, volume and density or developing a dynamic model with the soil properties and complex weather conditions [12,13]. However, these existing strategies have proved to be time consuming and heavily relied on soil and plant tests. Due to the limitations of traditional yield prediction methods, developing a rapid, nondestructive, and convenient method to estimate yields of cabbage timely would be beneficial for making management decisions and guiding fertilizer application.

Remote sensing technologies have been widely used in agriculture for precise management, nutrition diagnosis and in-season yield prediction [14]. Many plant indices based on multispectral sensors, the simple ratio (SR), ratio vegetation index (RVI), perpendicular vegetation index (PVI) etc., have shown their ability to accurately characterize plant physiological indexes, such as leaf area, plant nitrogen response and biomass [15,16,17]. Among these indicators, the normalized difference vegetation index (NDVI) value obtained by the Greenseeker active sensor has proven to be effective in predicting in-season yield of many crops [18,19]. The NDVI measurements are commonly used to characterizing the nitrogen condition or biomass development of plants, sometimes also applied for gaining regarding other nutritional monitoring in field crops of elements like potassium and phosphorus [20,21]. The Greenseeker hand-held optical sensor is a portable and convenient crop research and consulting tool that provides useful data to monitor plant status [22]. In general, the Greenseeker sensor emits wavelength at 671 ± 6 nm and 780 ± 6 nm to the plant canopy by its active light source, which the light reflectance of plants is measured based on light emitted from the sensor itself rather than from ambient light, and then the NDVI value is calculated by the reflectance at the specific region [23]. Studies have shown that the canopy reflectance to visible light (400–700 nm) was primarily dependent on the chlorophyll content in the palisade layer of the leaf and the NIR (near infrared) reflectance depended upon the structure of the mesophyll cell and the cavities between cells [24,25]. In a previous study, Olfs et al. (2005) found that the visible reflectance decreased while NIR reflectance increased due to N supplement [26]. Therefore, the measured NDVI values at nutrient deficient region were less than nutrient sufficient region, and the NDVI index could characterize the nitrogen status and plant biomass, which can be used to predict potential yield. In previous studies, the NDVI values obtained by the Greenseeker hand-held optical sensor have been proved to possess the potential to predict in-season yield of many crops, such as winter wheat, corn, rice and so on [27,28,29]. Ruan et al. (2001) showed that the NDVI measurements had a strong relationship with yield of winter wheat at Feekes growth stage 4 and 5 [19]. In corn, the NDVI value obtained by the Greenseeker at the V8 leaf stage has a strong relationship (R^2^ = 0.77) with harvested grain yield [30]. However, cabbage or other vegetables often deliver some unique characteristics, such as dispersed distribution, various varieties, short-term growth cycle and complicated canopy architecture; and few studies have directly examined the practical utilization of Greenseeker hand-held optical sensor in vegetable production systems. Limited studies such as Sanderson et al. (2012) found that Greenseeker had the potential to be an in-situ carrot health assessment tool and Dunn et al. (2015) reported that the NDVI measurements of ornamental kale showed good correlations with leaf N content [31,32]. Nevertheless, these studies didn’t build any yield prediction or nitrogen recommendation algorithm for vegetable. Therefore, predicting yield of cabbage and vegetable during the growing season is urgently needed for making appropriate production decisions and promoting precision management. Consequently, in-season yield prediction would be beneficial for improving yields and returns under particular conditions [30,33,34].

Given the paucity of information using sensors in yield prediction of cabbage and other vegetables, evaluating the potential of in-season yield prediction of cabbage using Greenseeker hand-held sensor is a promising step in developing a novel yield estimation approach for cabbage and other vegetables. In this work, a field experiment with two typical cabbage cultivars (Jianbao and Pingbao) in southeast China was conducted in 2014–2016, and the canopy reflectance characteristics was obtained by a Greenseeker hand-held sensor at each growth stage. The objectives of this study were to: (i) assess the relationship between NDVI measurements and yields of cabbage at different stages and identify the optimum growth stage for making reliable yield predictions; (ii) evaluate the improvement to yield forecasting accuracy by including the days from transplanting when GDD > 0 (DFP) and cumulative growing degree days (CGDD) values in regression models and (iii) establish the integrated quantitative relation to predict in-season yield of cabbage with different cultivars among years.

## 2. Materials and Methods

### 2.1. Site Description

**Figure 1:**
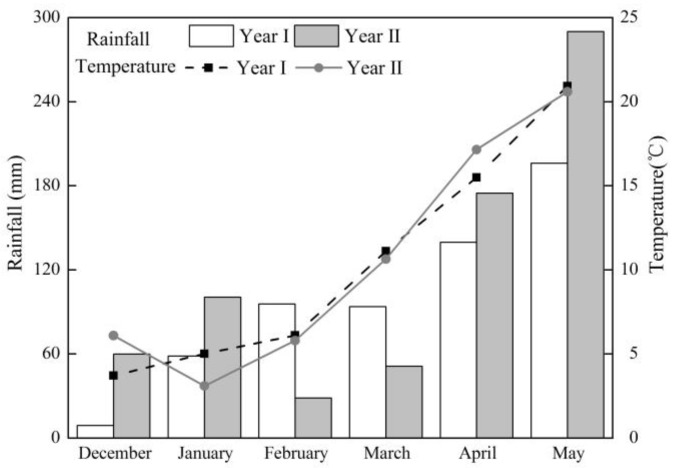
Monthly precipitation (mm) and average air temperature (°C) at the experiment site from December to May in cropping Year I (from 2014 to 2015) and Year II (from 2015 to 2016).

The two-year field experiment was conducted in 2014 and 2015 (Year I) and 2015 and 2016 (Year II) growing seasons (December to May) in a new vegetable field (paddy rice was the previous crop in 2013) in Yixing (31°16′ N, 119°54′ E), Jiangsu Province, which is located in the center of the Taihu Lake Region in southeastern China. The cabbage cultivar used in Year I was Jiangbao (JB), and the two cultivars used in Year II were Jianbao (JB) and Pingbao (PB). The JB cultivar used in this study is a common cultivar in this region, and have been widely planted for its special flavor. The region has a sub-tropical monsoon climate and the mean annual air temperatures and rainfall were 15.7 °C and 1177 mm, respectively. The distribution of precipitation and temperature during the experiment period is shown in Figure 1. The soil at the experiment site was classified as hydroagric Stagnic Anthrosol with a pH (H_2_O) of 6.25, electrical conductivity (EC) of 0.48 mS·cm^−1^, and total N and C content was 1.56 and 14.5 g·kg^−1^ soil, respectively [35].

### 2.2. Experiment Design

The experiment had four chemical N fertilizer rates. The four chemical N fertilizer treatments received 0, 80, 140, and 200 kg·N·ha^−1^ as urea, and are designated as N1, N2, N3, and N4, respectively. A basal does of 68 kg·P_2_O_5_·ha^−1^ and 68 kg·K_2_O·ha^−1^ from chemical fertilizer and 45 kg·N·ha^−1^ from manure was applied to cabbage at the time of transplanting. The urea-N levels of each treatment were applied either at 0, 75–80 and 105–110 DAT in 20%, 30% and 50% proportion split does, which was in accordance with the fertilizer requirement pattern of cabbage during growth stages [36,37]. The experiment was organized in a randomized complete block design with three replications, 33.6 m^2^ (7 m × 4.8 m) for each block. Cabbage seedlings were grown in a small nursery first and then transplanted at the two-leaf stage into the experimental plots and grown for 145 days. Cabbage was transplanted on 18th December, 2014 in Year I and 20th December, 2015 in Year II. Weeds were controlled by applying pre-emergence herbicide before transplanting, and weeds that escaped these treatments were removed manually at 80 DAT. The field was furrow irrigated immediately after transplanting and around 30 and 80 DAT at a water quantity of approximately 0.3 m^3^/plot each time. In addition, 24 additional cabbage plots (4 m^2^ each) located at about 200 m far from the experiment site were selected as validation plots in Year II, and the variety of cabbage in the validation field was JB cultivar and the daily management was the same as our experimental plots.

### 2.3. Data Collection

**Figure 2:**
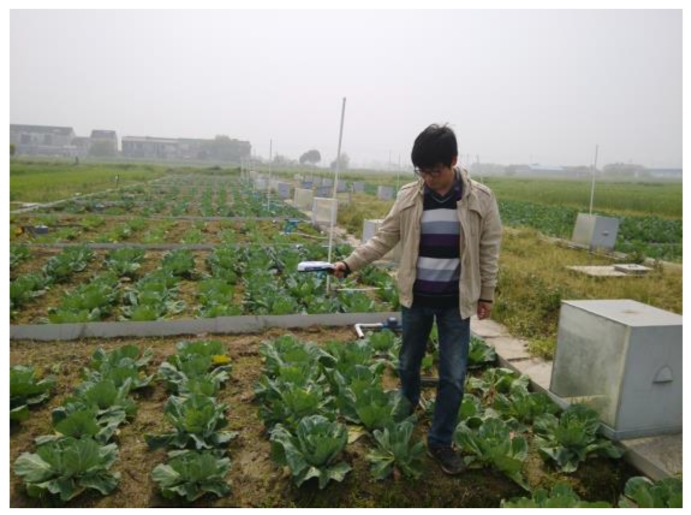
Picture of Greenseeker taking measurements over the canopy of cabbage.

The canopy spectral reflectance characteristics of cabbage were obtained by a Greenseeker^TM^ (N-Tech Industries, Inc., Ukiah, CA, USA) hand-held sensor. The NDVI values were collected at 20, 50, 70, 80, 90, 100, 110, 120, 130, and 140 DAT with the sensor positioned 60 cm above the crop canopy. The NDVI was collected at about 9:00–10:00 a.m. each time and the average of 4 measurements from each plot was reported. Picture of Greenseeker taking measurements over the canopy of cabbage is shown in Figure 2. Yield of each plot was obtained at harvest 145 DAT. 

In addition, the NDVI/DFT ratio was calculated by dividing NDVI by DFT (number of days from transplanting to sensing when GDD (growing degree days) > 0).

The NDVI/CGDD ratio was calculated by dividing NDVI by CGDD (cumulative growing degree days) from transplanting to sensing when GDD > 0. 

GDD = (T_max_ + T_min_)/2–5 °C, T_max_ and T_min_ represent daily maximum and minimum temperature, respectively. 5 °C is the base growing temperature for cabbage. 

### 2.4. Statistical Analyses

The data were subjected to analysis of variance (ANOVA) and Duncan multiple-comparison test to determine the differences among treatments with SPSS (ver. 20.0 for Windows, SPSS Inc., Chicago, IL, USA). Pearson correlation coefficients were also calculated with the use of SPSS program. The regression analyses and models were built with Matlab (The MathWorks Inc., Natick, MA, USA). All graphs were plotted with Origin 8.5 (OriginLab Corporation, Northampton, MA, USA).

## 3. Results

### 3.1. Yield Responses to N Rates

**Figure 3:**
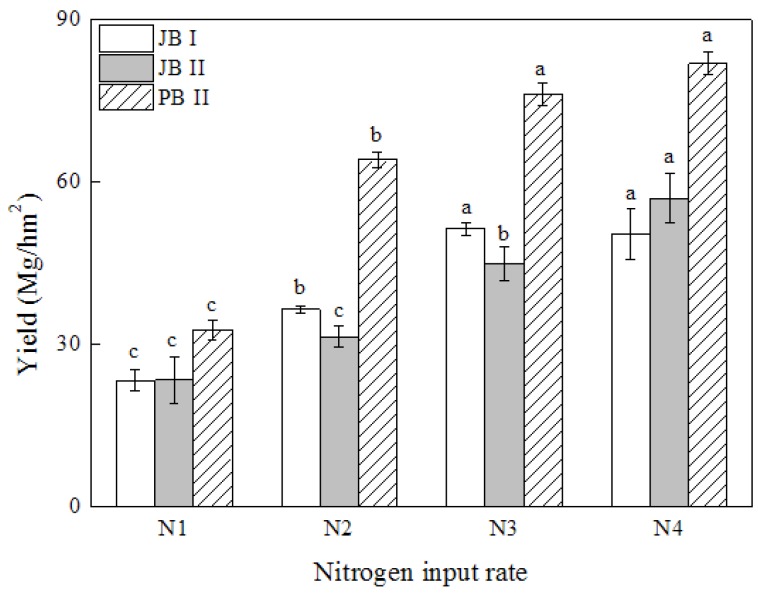
Cabbage yields of JB cultivar in year I (JB I) and II (JB II) and PB cultivar in year II (PB II) response to different chemical fertilizer N application rates. Treatments N1, N2, N3 and N4 received 0, 80, 140, and 200 kg·N·ha^−1^ in the growth season, respectively. Different letters indicate significantly differences among different N input rates of the same cultivar at the *p* < 0.05.

Cabbage yields at different fertilizer N application rates are shown in Figure 3. The yields of both cabbage cultivars were significantly improved by N fertilizer application in both years. Compared with the control (N1), the yields of JB cultivar under N2, N3 and N4 treatments were increased by 44.9%, 105.4%, and 128.8%, respectively. The yields of JB were similar in year I and II except for N2 and N3 which had slightly lower yields in year II. As presented in Figure 3, the yields of PB cultivar under each N treatment were found significantly higher than that of the JB cultivar. Although the yield changing pattern was similar for both cultivars at different N rates, the actual yield gap of the two cultivars was rather significant; and the yield of PB cultivar was about 39.6%, 88.8%, 58.1%, and 52.7% higher than that of JB cultivar at the 4 equal N application rates, respectively. For both cultivars, yields increased as N rate increased from N1 to N3, but no further increase was observed for N4, suggesting that the N3 was the optimum rate for cabbage production. Cabbage yields significantly responded to fertilizer N application rate up to 140 kg·N·ha^−1^ and the PB cultivar in our experiment had greater yield potential than the JB cultivar. 

### 3.2. Relationships between NDVI Measurements and Cabbage Yields

The Pearson correlation analysis results of NDVI measurements and yield of cabbage with JB cultivar in year I and II and PB cultivar in year II are shown in Table 1. The “Pearson r” value showed that NDVI measurements at different growth stages had a significant relationship with the yield of cabbage in both experiments. In JB I and II growth seasons, the “Pearson r” values ranged from 0.250 to 0.940 and 0.442 to 0.886, respectively, and the highest correlation coefficient was reached at 110 DAT, for both years. In PB II seasons, the “Pearson r” values were almost zero at the early growth stage, and there was a positive relationship between the measured NDVI values and in-season yield of cabbage after 100 DAT (*p* < 0.01). The highest “Pearson r” value of PB cultivar was achieved at 130 DAT while the correlation coefficient was relatively stable after 110 DAT. There existed similar trends for the “Pearson r” value over time during growth seasons for JB I and II and PB II, and the correlation coefficient was relatively low at the early growth stage, and then the robust relationship was achieved at about 110 DAT. The “Pearson r” value remained unchanged or decreased thereafter. 

To quantify the relationship between NDVI measurements and yield of cabbage, exponential, linear and quadratic functions were used to regress cabbage yields with NDVI measurements collected during growth seasons, and the significance of each regression model was evaluated using the determination coefficient and an F-test for tracking the fitness (Table 2). There was no significant difference among the regression analyses results of the three equation types. It revealed that NDVI measurements before 80 DAT exhibited very low coefficient of determination with cabbage yields of JB cultivar in year I. The highest value of determination coefficient with the three types of regression equation all achieved at 110 DAT at around 0.88. Additionally, the regression analysis couldn’t make satisfactory prediction of in-season yield with NDVI readings of JB and PB cultivar in year II before 100 DAT. In JB II growth season, the R^2^ improved to about 0.80 and the best prediction effect was observed at 110 DAT with the exponential equation, and it declined to around 0.40 at 140 DAT. The highest R^2^ between NDVI and cabbage yield of PB cultivar was obtained at 120 DAT, but there was no significant difference between the R^2^ values at 110 DAT and 120 DAT. Therefore, the earliest timing to make satisfactorily yield prediction was at 110 DAT. Thus, the common exponential, linear and quadratic regression models could make adequate prediction of cabbage yield and the NDVI measured at 110 DAT was the only important parameter in predicting in-season yield of cabbage. 

### 3.3. Predicting Cabbage Yields Using Measured NDVI at the Optimum Time

**Figure 4:**
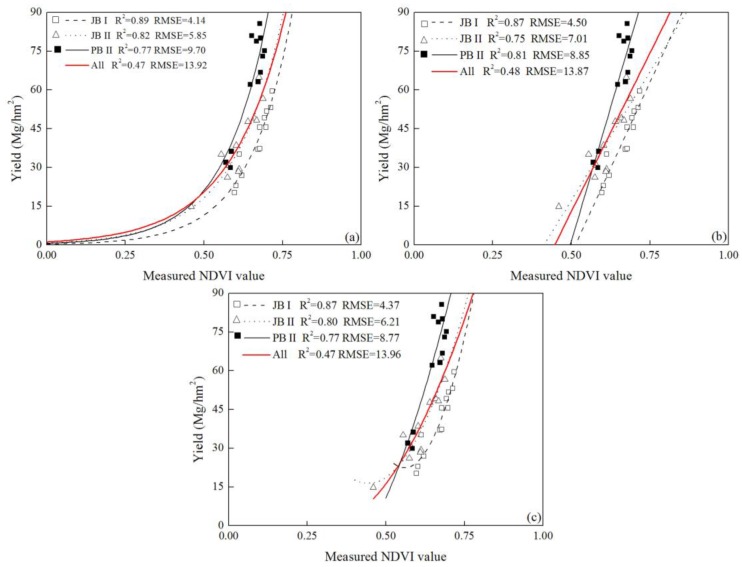
Plots of the relationship between observed cabbage yield and NDVI (normalized difference vegetation index) measurements for all cultivar-years (JB cultivar in year I and II (JB I and JB II) and PB cultivar in year II (PB II)); (**a**) exponential, (**b**) linear and (**c**) quadratic equation).

The results summarized in the regression analyses showed that the 110 DAT was the most appropriate time for yield prediction of cabbage. The fitting curves of measured NDVI values and cabbage yield at this stage using exponential, linear and quadratic fitting types are presented in Figure 4. In each growing season, the measured NDVI values could well explain the yield variation and accurately predict in-season yield of JB cultivar in year I and II and PB cultivar in year II, with R^2^ values ranged from 0.87–0.89, 0.75–0.82 and 0.77–0.81, respectively. However, the result of using one integrated equation to predict in-season yield for both years and cultivars was not satisfactory, since the R^2^ value was only 0.47–0.48 for the three fitting types, and the RMSE value was between 13.87 and 13.96, suggesting the equations couldn’t make adequate prediction of in-season yield of cabbage cross cultivars and years. As shown in Figure 4, there was a significant deviation of data points from the combined curve. The deviation of JB cultivar in year I and II was much greater than that of the JB and PB cultivar in year II. Thus, the combined fitting curves couldn’t reflect the NDVI-yield response of JB cultivar in different years, which was partly influenced by different weather conditions between years (Figure 1).

### 3.4. Modification of the Yield Prediction Equation with DFT and CGDD Values

**Figure 5:**
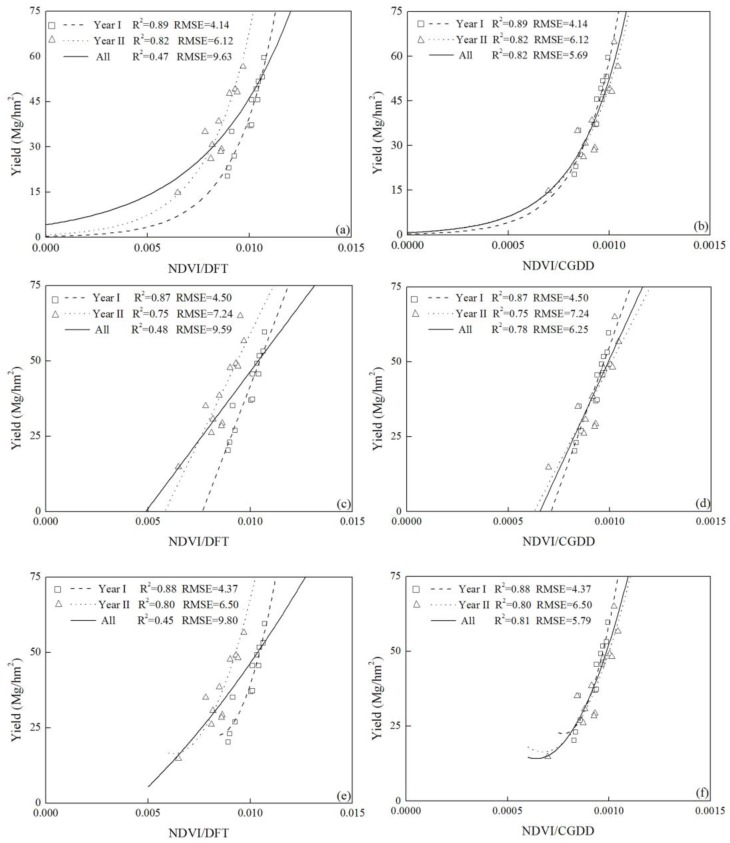
Using the DFT and CGDD values to modify the exponential: (**a**) and (**b**), linear: (**c**) and (**d**), and quadratic: (**e**) and (**f**) yield prediction equations, respectively. The data presented in the figure was obtained from the result of JB cultivar in year I and II. The DFT and CGDD values represent the number of days from transplanting to sensing where GDD (growing degree days) > 0) and the cumulative growing degree days from transplanting to sensing, respectively.

It should be noted that the different weather conditions between years contributed to the variation of the integrated yield prediction curves to some degree. Therefore, we attempted to remove some environmental influences by including DFT and CGDD values in the yield prediction equation, and some changes were observed in the accuracy of the three yield prediction equations (Figure 5). The DFT values of year I was 67 compared to 71 in year II, while the CGDD values of year I and II were 721.8 and 658.8, respectively. The adjustment with NDVI/DFT values did not significantly improve the yield prediction model and the R^2^ value remained the same (0.47), and none of the three equations could explain some of the yield variations. However, the prediction was greatly improved by incorporating the CGDD values. The NDVI/CGDD value based yield prediction curves could explain 78–82% of the yield variation. Among the three types of equations, the exponential function performed the best in predicting the in-season yield of cabbage, and the coefficient of determination and RMSE was 0.82 and 5.69, respectively. The linear function with NDVI/CGDD values was slightly less predictable than the exponential one, and the former had a R^2^ value and RMSE of 0.78 and 6.25, respectively. It is clear that the CGDD based modification could significantly improve the accuracy of the yield prediction equations, and the exponential function performed better than the linear and quadratic one.

### 3.5. Model Validation

**Figure 6:**
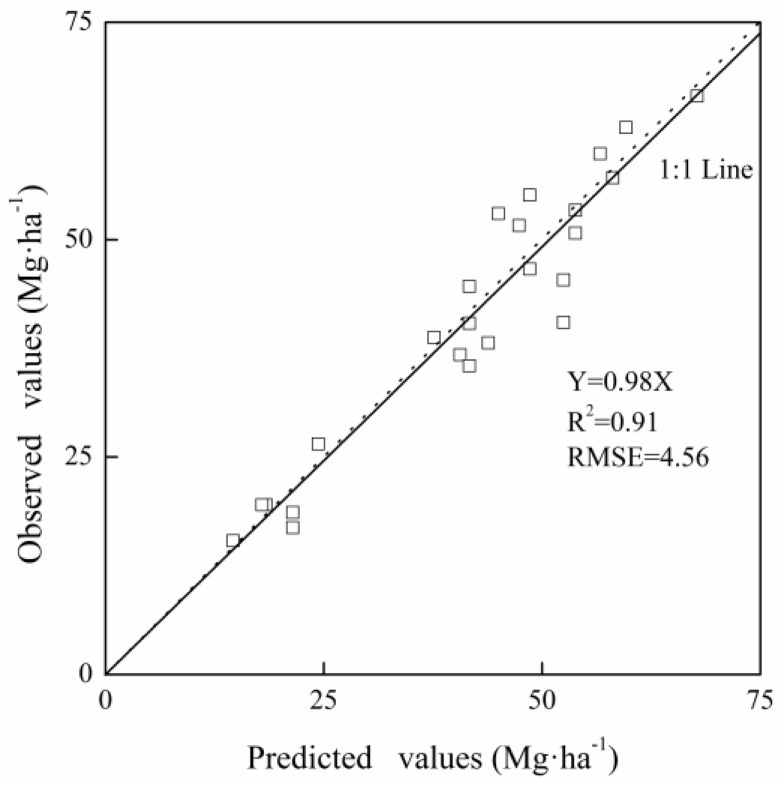
Relationship between the observed and predicted yields of the exponential yield prediction model for estimating in-season yield of cabbage.

The exponential equation developed from modified NDVI measurements with CGDD values at 110 DAT could explain the in-season yield variation for cabbage satisfactorily, and the integrated yield prediction model for all cultivar-years is shown in Table 3. The combined equation had a high coefficient of determination (0.80) and low RMSE (8.71). To validate the reliability of integrated in-season yield prediction model, we tested the model using an independent data set obtained from validation field planted with JB cultivar in year II near the experiment field. As shown in Figure 6, the predicted yields were highly correlated with the harvested yields (R^2^ = 0.91) in almost 1:1 relationship with a slope of 0.98. In general, the data points of the validation equation were scattered evenly around the 1:1 line. This confirms the integrated yield prediction model could predict yield of cabbage accurately.

## 4. Discussion

### 4.1. In-Season Prediction of Cabbage Yield

Crops significantly responded to N fertilizer application [38]. Our results showed that the N application rates (N2 and N3) also significantly increased the cabbage yield than the control. However, when the N application rate raised to N4 level no further increase in yield, and even a yield loss was observed at this rate (Figure 3). This is consistent with the findings that over applying N fertilizer would not increase yield but might lead to high N losses [39,40]. Thus, timely predicting yield in the growing season would guide N fertilizer application and achieve optimum economical yields. Greenseeker optical sensor has been successfully used in estimating yields of grain crops, and the preliminary and accurately estimation of yields would provide ponderable information for making decisions related to N management [41]. The various varieties and relatively complicated canopy architecture of cabbage resulted in the uncertainty of whether this precision tool would be used in in-season yield prediction. Lofton et al. (2012) found some difficulties for the yield prediction of sugarcane as its multi-year cropping cycle combined with the shorter growth period in Louisiana [27]. In this study, the Pearson correlation analysis showed that NDVI measurements had a significant positive relationship with the yields of cabbage in both experiments (Table 1), indicating that the handy Greenseeker sensor had a great potential to be used for cabbage yield prediction and N adjustment.

Similar to rice crop, the robust relationship was not sustained throughout the growth stages obviously (Table 1). At heading stage, the Greenseeker sensor indices of rice became saturated and consequently could not be used for estimation in-season yield, while at the early growth stages (tillering stage) the rice canopy was not closed but the soil and water background would have a strong influence on canopy reflectance [42,43]. Our experiment was conducted in an open vegetable field in southeastern China. At the early stage (before 90 DAT), the temperature was relatively low and growth rate was minimal (Figure 1). Therefore, the nutrient uptake was relatively low and cabbage biomass was not fully developed by this stage. At the middle growth stage (around 110 DAT), the growth of cabbage picked up significantly, and it was the appropriate stage for topdressing N [44]. However, at later stage close to maturity, the relationship seemed stable but weak, because the cabbage entered into the heading stage and the canopy began to close. The sensing timing is crucial for predicting cabbage yield, as found for winter wheat, dry direct-seeded rice and corn as well [19,30,45].

### 4.2. Comparison of Various Yield Prediction Equations

Linear and non-linear regression analyses were commonly used in past studies to predict crop yields in-season [19,29,46]. In our study, no significant difference was found among the exponential, linear, and quadratic equations established. In addition, a similar change pattern was observed with the R^2^ values during the growing seasons (Table 2). By comparison and equation modification, the exponential equation showed a better performance than the linear and quadratic functions; thus, the exponential equation was selected to predict the in-season yield of cabbage (Table 3). Similar approaches were reported by Raun et al. (2001) and Teal et al. (2006) for winter wheat and corn, respectively [19,30]. As mentioned in previously cases, an exponential yield prediction model generally used in the occasion that the growing rate of crops at a giving time is in proportion to the remaining growth [47,48]. Given the growing characteristics of cabbage at the 110 DAT, the exponential model built in our study was suitable for estimating the growth rate of cabbage at this growth stage.

### 4.3. Effect of Climatic Variability on the Yield Prediction Model

Our results suggested that the accuracy of integrated yield prediction model with NDVI measurements was not satisfactory, and there was a wide disparity between the data points of JB cultivar in year I and year II (Figure 4). The climate differences between years and locations may influence the process of in-season yield prediction of cabbage [19,46]. DFT and CGDD values were considered the effective growth days and cumulative temperature across the growing season, respectively [27]. However, as demonstrated, adjusting NDVI measurements with CGDD values increased the accuracy of yield prediction while the DFT values did not (Figure 5). These results were consistent with the earlier work for sugarcane [27] and winter wheat [49]. Those researchers also found modification with CGDD values provided a better estimation of temperature throughout growth season compared with DFP values. However, in dry direct-seeded rice [45] and corn [30], the use of CGDD modification did not improve the accuracy of yield prediction. This was partly due to the different biomass, crop characteristics and environmental conditions, but modification with CGDD broadened the utilization range of yield prediction in various climates and locations as well.

### 4.4. Effect of the Cultivar Difference on the Yield Prediction Model

Various phenotypes acted differently to ambient environment [50], and this was the potential reason for the different behavior of the two cultivars used in our study. PB cultivar in our experiment achieved higher yields and NDVI measurements than JB did (Figure 3), which was probably due to the diverse canopy architecture and heading styles between the two cabbage cultivars and may be also influenced by the soil background fertility levels at some degree [51]. The PB cultivar had wider leaves and larger surface area [27]. These results were in agreement with the finding of Wang et al. (2013) in rice, where the multiple cultivars responded differently to nitrogen input and had disparate spectral characteristics [52]. In our study, the difference between cultivars was smaller than the discrepancy of one cultivar between years (Figure 4). And in year II, the scatters of PB cultivar were gathered at the top of the curve, while the data points of JB cultivar were gathered at the bottom of the curve, but the tendency of the two curves was quite similar. As demonstrated by the integrated equation, the coefficient of determination was 0.80, comparing with the results in winter wheat, sugarcane, corn and dry direct-seeded rice, where the yield prediction equations explained 83%, 48%, 77% and 63% of the yield variation, respectively. The result in our study was almost same or even much better than the prediction effect in other crops, therefore, the yield of different cultivars could be well explained (Table 3) [19,27,30,45]. These results implied that cabbage yield could be accurately predicted in season although there are differences between the two cultivars and temporal climatic variability.

## 5. Conclusions

In our two-year field experiment, the Pearson and regression analyses showed that the NDVI measurements obtained by the Greenseeker hand-held optical sensor at 110 DAT were significantly associated with the yield of JB cultivar. Consideration of the CGDD values was important in increasing the accuracy of prediction. The exponential equation is more superior in yield prediction than linear or quadratic equations, and the integrated exponential yield model explained 80% of the yield variations with the validation of JB cultivar could make satisfied estimation between observed and predicted yields. Therefore, the Greenseeker hand-held optical sensor is an accurate and convenient tool to determine the in-season yield of JB cultivar, and this comparatively simple method would provide an alternative approach for farmers to estimate yield potential of cabbage in practice and make adjustment for N management when necessary. Further work is needed to generalize the results to other varieties of cabbage and vegetables and to develop a better fertilizer recommendation system for cabbage and other vegetables.

## Figures and Tables

**Table 1:** Pearson correlation analysis of the NDVI measurements and yield of JB cultivar in year I and II and PB cultivar in year II.

DAT (Day)	JB I	JB II	PB II
20	0.250	0.442	−0.273
50	0.677 *	0.566	−0.038
70	0.452	0.573	0.109
80	0.807 **	0.612 *	−0.389
90	0.888 **	0.661 *	0.110
100	0.807 **	0.781 **	0.726 **
110	0.940 **	0.886 **	0.907 **
120	0.895 **	0.831 **	0.951 **
130	0.761 **	0.725 **	0.960 **
140	0.891 **	0.687 *	0.956 **

Note: *: significant correlation at the 0.05 level, **: significant correlation at 0.01 level. The correlation coefficients were calculated at each sampling date *n* = 12 for each cabbage cultivar at every sampling time.

**Table 2:** The correlation coefficients (R^2^) between NDVI measurements and cabbage yield of JB cultivar in year I and II and PB cultivar in year II using three different equations.

DAT	Cultivar								
	JB I			JB II			PB II		
	E ^1^	L ^2^	Q ^3^	E	L	Q	E	L	Q
20	0.03	0	0	0.19	0.11	0.05	0.07	0	0
50	0.36 *	0.40 *	0.46 *	0.25	0.25	0.17	0.01	0	0
70	0.20	0.13	0.03	0.24	0.26	0.21	0.01	0	0
80	0.54 **	0.62 **	0.68 **	0.31 *	0.31 *	0.24	0.16	0.07	0.05
90	0.76 **	0.77 **	0.74 **	0.38 *	0.38 *	0.31 *	0.01	0	0
100	0.58 **	0.62 **	0.58 **	0.59 **	0.57 **	0.52 **	0.49 **	0.48 **	0.47 **
110	0.89 **	0.87 **	0.87 **	0.82 **	0.75 **	0.80 **	0.77 **	0.81 **	0.77 **
120	0.84 **	0.78 **	0.86 **	0.71 **	0.66 **	0.66 **	0.89 **	0.90 **	0.89 **
130	0.56 **	0.54 **	0.54 **	0.48 **	0.48 **	0.42 *	0.86 **	0.91 **	0.94 **
140	0.85 **	0.77 **	0.86 **	0.39 *	0.42 *	0.41 *	0.86 **	0.91 **	0.91 **

^1^ Represented the exponential equation, and formula $y_{yield}=a\times e^{b\times x_{NDVI}}$ was used; ^2^ represented the linear equation, and formula $y_{yield}=a\times x_{NDVI}+b$ was used; ^3^ represented the quadratic equation, and formula $y_{yield}=a\times x_{NDVI}{}^{2}+b\times x_{NDVI}+c$ was used, a and b are regression parameters in each equation. *: Means significant correlation at the *p* < 0.05 level, **: significant correlation at the *p* < 0.01 level.

**Table 3:** Regression coefficients (a and b), coefficient of determination (R^2^) and root mean square error (RMSE) for the cabbage in-season yield prediction models.

	Plant Index	Monitoring Time	R^2^	Regression Parameters ^a^		RMSE
				a	b	
Yield prediction model	NDVI/CGDD	110 DAT	0.80	0.316	5230	8.71

^a^ Regression parameter, $\mathrm{in}-\mathrm{season}\;\mathrm{yield}=a\times e^{b\times index\;value}$. The integrated yield prediction model was built from the data obtained with JB cultivar in year I and II and PB cultivar in year II.

## Abbreviations

NDVI: normalized difference vegetation index
DAT: days after transplanting
DFP: days from transplanting when GDD > 0
GDD: growing degree days
CGDD: cumulative growing degree days
RMSE: root mean square error
